# FLAIR-based radiomics signature from brain-tumor interface for early prediction of response to EGFR-TKI therapy in NSCLC patients with brain metastasis

**DOI:** 10.3389/fcell.2025.1525989

**Published:** 2025-05-14

**Authors:** Chunna Yang, Yiyao Sun, Mingchen Jiang, Ying Fan, Yanjun Hu, Qianhui Zhang, Yan Zhang, Yan Wang, Xiran Jiang, Zekun Wang, Zhiguang Yang, Bo Sun, Wenyan Jiang

**Affiliations:** ^1^ School of Intelligent Medicine, China Medical University, Shenyang, Liaoning, China; ^2^ Center for Biomedical Engineering, School of Information Science and Technology, Fudan University, Shanghai, China; ^3^ Department of Medical Imaging, Cancer Hospital of China Medical University, Liaoning Cancer Hospital and Institute, Shenyang, Liaoning, China; ^4^ Public Health Supervision Technology Department, Liaoning Provincial Center for Disease Prevention and Control, Liaoning, China; ^5^ Department of Radiology, Shengjing Hospital of China Medical University, Shenyang, China; ^6^ Department of Radiology, The First Affiliated Hospital of Dalian Medical University, Dalian, China; ^7^ Department of Scientific Research and Academic, Cancer Hospital of China Medical University, Liaoning Cancer Hospital and Institute, Shenyang, China

**Keywords:** T2-FLAIR, brain metastasis, TKI therapy, MRI, radiomics

## Abstract

**Objectives:**

Evaluating response to epidermal growth factor receptor (EGFR)-tyrosine kinase inhibitors (TKIs) is crucial in non-small cell lung cancer (NSCLC) patients with brain metastases (BM). To explore values of multi-sequence MRI in early assessing response to EGFR-TKIs in non-small cell lung cancer (NSCLC) patients with BM.

**Approach:**

A primary cohort of 133 patients (January 2018 to March 2024) from center one and an external cohort of 52 patients (May 2017 to December 2022) from center two were established. Radiomics features were extracted from 4 mm brain-tumor interface (BTI) and whole BM region across T1-weighted contrast enhanced (T1CE) and T2-weighted (T2W) and T2 fluid-attenuated inversion recovery (T2-FLAIR) MRI sequences. The most relevant features were selected using the U test and least absolute shrinkage and selection operator (LASSO) method to develop the multi-sequence models based on BTI (RS-BTI-COM) and BM (RS-BM-COM). By integrating RS-BTI-COM with peritumoral edema volume (VPE), the combined model was built using logistic regression. Model performance was evaluated using the area under the ROC curve (AUC), sensitivity (SEN), specificity (SPE) and accuracy (ACC).

**Main Results:**

The constructed RS-BTI-COM demonstrated a higher association with early response to EGFR-TKI therapy than RS-BM-COM. The combined RS-BTIplusVPE, incorporating BTI-based radiomics features and VPE, exhibited the highest AUCs (0.843–0.938), SPE (0.808–0.905) and ACC (0.712–0.875) in the training, internal validation, and external validation cohort, respectively.

**Significance:**

The study developed a validated non-invasive model (RS-BTIplusVPE) based on integrating BTI-based radiomics features and VPE, which showed improved prediction of EGFR-TKI response in NSCLC patients with BM compared to tumor-focused models.

## 1 Introduction

Lung cancer has become the leading cause of cancer deaths globally, accounting for more than 25% of all cancer deaths each year (Brainard and Farver, 2019). Non-small cell lung cancer (NSCLC) is the most common subtype of lung cancer, accounting for approximately 85% of all lung cancers (Gridelli et al., 2015). Studies have shown that approximately 40% of NSCLC cases have distal metastases at the time of diagnosis (Little et al., 2007; Schuchert and Luketich, 2003). Brain or central nervous system as the most common site of metastasis in NSCLC with the incidence rate of 40%–50% (Sørensen et al., 1988; Yawn et al., 2003). The development of brain metastasis (BM) would severely lead to poor prognosis for NSCLC patient, with the median overall survival ranging between 2–9 months (Peters et al., 2016).

In recent years, epidermal growth factor receptor tyrosine kinase inhibitor (EGFR-TKI) therapy has been shown to display superior efficacy compared to standard chemotherapy (Ettinger et al., 2017), and has profoundly impacted the therapeutic landscape of NSCLC (Yuan et al., 2019; Lynch et al., 2004). However, approximately one-third of TKI-treated NSCLC patients do not benefit from the EGFR-TKI therapy. Their tumors continue to have rapid progression despite the treatment (Kawaguchi et al., 2014; Spigel et al., 2017), which indicates that many patients may be at risk for rapid deterioration of clinical symptoms, delayed treatment, poor prognosis and even death (Yang et al., 2013).

The Response Evaluation Criteria for Solid Tumours (RECIST 1.1) provides an objective, standardized method for assessing the efficacy of EGFR-TKIs (Eisenhauer et al., 2009), and requires the visual assessment of the tumor size based on radiological imaging (Mayerhoefer et al., 2020). Magnetic Resonance Imaging (MRI) has been widely and routinely used to assess response to EGFR-TKI. However, it relies heavily on visual assessment, making it highly subjective and less accurate (Mayerhoefer et al., 2020; Chetan and Gleeson, 2021). This limitation arises from the lack of preoperative specific biomarkers in MRI imaging that can identify patients who would benefit from EGFR-TKI therapy. Previous studies (Hsiao et al., 2020; Guo et al., 2020; Li et al., 2015) have explored molecular markers for predicting EGFR-TKI efficacy, including soluble cadherin-3, genetic alterations, and COX-2 serum levels, but these markers have not been widely validated. For PET/CT imaging, recent studies (Zhu et al., 2022; Agüloğlu et al., 2022; Shao et al., 2020) have shown its potential in predicting treatment response and progression-free survival, but its role in predicting the response to EGFR-TKI therapy in BM remains unclear. Therefore, a novel method that enable early predict response to EGFR-TKI before treatment is essential for the development of an appropriately individualized treatment regimens.

Radiomics have been addressed to the field of precision medicine for making individual therapeutic decisions based on medical imaging data (Sharpton et al., 2014). Previous studies have shown that the development of radiomic can be helpful to identify valuable features associated with EGFR-TKI response, enabling non-invasive assessment of therapeutic efficacy of EGFR-TKI (Chetan and Gleeson, 2021; Sharpton et al., 2014; Zhang et al., 2023; Song et al., 2020; Mu et al., 2020; Song et al., 2018; Bera et al., 2022). Fan’s group recently conducted radiomic studies investigating the role of brain MRI on BM for assessment of response to EGFR-TKI (Fan et al., 2023a; Fan et al., 2022). However, the studies only evaluated T1-weighted contrast enhanced (T1CE) and T2-weighted (T2W) MRI, and neglected the potential value of brain T2 fluid-attenuated inversion recovery (T2-FLAIR) sequence. The T2-FLAIR sequence can effectively suppress cerebrospinal fluid signals and highlight adjacent lesions, and has been widely used in the diagnosis of central nervous system diseases (Tha et al., 2009). Moreover, the T2-FLAIR can quantify the degree of peritumoral edema and inflammation in BM (Zakaria et al., 2021). Previous reports have demonstrated that features derived from T2-FLAIR are strongly correlated with gene mutation status (Wang et al., 2021) and immune responses (Madi et al., 2022). While, the role of T2-FLAIR MRI on BM for predicting EGFR-TKI therapeutic efficacy has not yet been investigated.

Besides, the BM has a unique microenvironment (Eichler et al., 2011). The interface between brain parenchyma and tumor (brain-to-tumor interface, BTI) represents the area that metastatic tumor cells interact with endocranial brain cells and patient’s immune system (Berghoff et al., 2013). This region has garnered significant interest in recent years, with BTI-focused radiomics proven to effectively indicate the extent of brain invasion (Xiao et al., 2021b; Joo et al., 2021; Li et al., 2021) and assess tumor grading (Zhao et al., 2024). This underscores the potential utility of the BTI in computer-aided diagnosis of BM. This study aims to evaluate the value of the MRI image of BM for predicting response to EGFR-TKI therapy based on the BTI area in NSCLC patients. Our findings and developed models were further tested with an external validation set.

## 2 Materials and method

### 2.1 Patients

**FIGURE 1:**
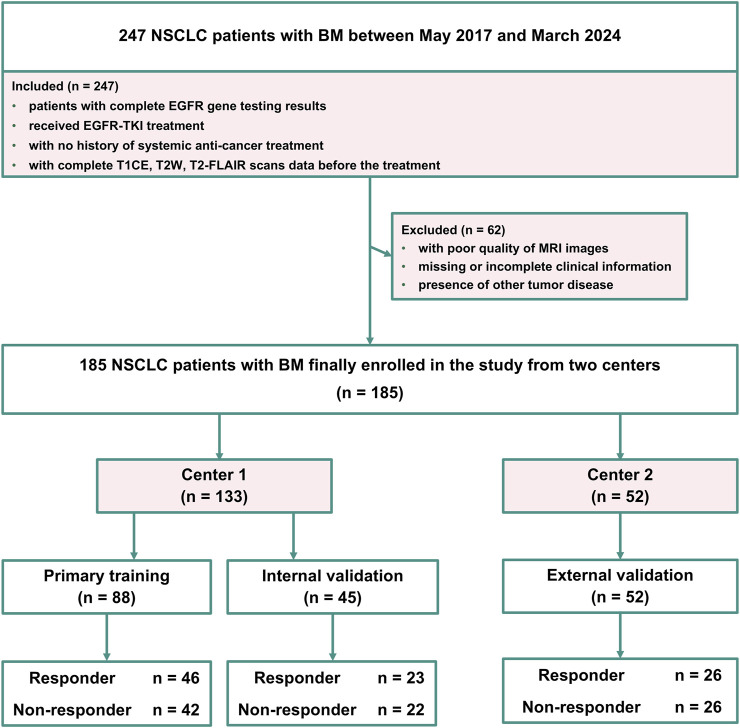
Flowchart of the patients included in this study.

The MRI data of patients with BM included in this retrospective study were obtained with the approval of the local ethics committee (number: 20,220,659), and all patient data were anonymized to ensure confidentiality. Center one enrolled 133 patients with EGFR-mutant NSCLC with BM from January 2018 to March 2024, forming the primary cohort used to construct the training set and internal validation set. Center two included 52 patients with EGFR-mutant NSCLC with BM from May 2017 to December 2022, serving as an external validation cohort. The inclusion criteria for all the patients were as follows: (i) patients with complete EGFR gene testing results, (ii) received EGFR-TKI treatment, (iii) with no history of systemic anti-cancer treatment, and (iv) with complete T1CE, T2W and T2-FLAIR scans data before the treatment. Patients were excluded if they met any of the following criteria: (i) with poor quality of MRI images, (ii) missing or incomplete clinical information, or (iii) presence of other tumor disease. The response to EGFR-TKI treatment was evaluated using the RECIST 1.1 criteria (Therasse et al., 2000; Therasse et al., 2006). The primary cohort (Center 1) was divided into a training cohort and an internal validation cohort in a stratified 2:1 ratio. Stratified random sampling was performed using the strata function in R with the srswor method, ensuring random assignment without replacement. The external cohort (Center 2) was utilized for independent external validation of the developed radiomics models. Figure 1 shows the patient inclusion flowchart.

### 2.2 MRI protocol and tumor delineation

T1CE, T2W, and T2-FLAIR sequences were acquired on a 3.0 T scanner for image analysis. Details of the brain MRI scanning devices and parameters were listed in Supplementary Material 1. Regions of interest (ROIs) segmentation was performed by the first radiologist (Y.J.H., with 4 years’ experience), who had no knowledge of the patient’s clinicopathological information, except for the tumour location. ROIs of the BM and peritumoral edema area (PEA) in the brain were manually drawn using the open-source software ITK-SNAP (version 3.6.1, www.itksnap.org). All manual depictions were validated by the second radiologist (Z.G.Y., with 19 years’ experience).

### 2.3 BTI generation

We utilized a semi-automatic segmentation algorithm to delineate the BTI region. In the Python software, the manually outlined BM contour was eroded by 2 mm inwards and dilated by 2 mm outwards, resulting in a final annular region with a width of 4 mm. After obtaining the ROI of the BTI region, we calculate the entropy value of the BTI region to quantify the entropy of the pixel intensity distribution within the region, reflecting its texture complexity and information content. The formula for calculating the entropy is shown in the Supplementary Material 2. The peritumoral edema volume (VPE) was computed based on the PEA that was demarcated by using the ITK-SNAP software. In summary, the three regions used in this study are: (i) BM, manually delineated for feature extraction and BM-based model development; (ii) PEA, the edema region surrounding the BM, manually delineated to calculate the value of VPE; and (iii) BTI, an annular region derived by expanding and contracting the BM contour through a python script, containing information about the interface between the PEA and the active tumor region of BM, used for feature extraction and BTI-based model development. The representative brain MR images of three different sequences are shown in the (Supplementary Figure S1).

### 2.4 Feature extraction

First-order, texture, shape and filter features were extracted from the manually segmented tumor regions and the BTI region. The first-order features can provide information about the overall brightness, contrast, and distribution of an image. The texture features include 24 Gray-level co-occurrence matrix, 14 Gray-level dependence matrix, 16 Gray-level size zone matrix and 16 Gray-level run length matrix. The shape features quantitatively describe the 2D size and shape of the ROIs. To obtained filtered features, the original images were filtered by using eight filters, which include Wavelet, square, squareroot, laplacian of Gaussian, logarithm, gradient, exponential, and local binary pattern 2D/3D, then used to calculate textural and first-order features. Finally, a total of 1,967 radiomics features were generated for each MR sequence (T1CE, T2W and T2-FLAIR). Imaging preprocessing and feature calculating were performed using the Pyradiomics package (version 3.0.1) according to a previous report (van Griethuysen et al., 2017).

### 2.5 Feature selection and model construction

To select highly correlated and minimally redundant features, we employed the following feature selection strategy: Firstly, we applied the Mann-Whitney U test to the features, where features with a p-value <0.05 were considered significantly different and retained for further screening. Secondly, the most predictive features were determined using the least absolute shrinkage and selection operator (LASSO) with ten-fold cross-validation (Tibshirani, 1997). Thirdly, we identified the most predictive features using logistic regression with stepwise selection based on Akaike Information Criterion (AIC) minimization, derived from the BTI and BM regions (Pan, 2001). Finally, we evaluated these most predictive features using intra-class correlation coefficient (ICC) (Leijenaar et al., 2013). We randomly selected thirty patients to assess the reproducibility of the selected features. Features with an ICC >0.80 were considered to have better reproducibility and were retained for further construction. More detailed information on the ICC calculation can be found in the Supplementary Material 1.

In the subsequent model construction process, we predicted the response to EGFR-TKI therapy using a logistic regression classifier, a widely accepted and effective machine learning classifier, implemented using the glmnet package in R (Bera et al., 2022). Specifically, the selected most predictivefeatures were used to develop radiomics models based on the BTI region, the combined radiomics signatures (RSs) named RS-BTI-COM was constructed by integrating all the features from T1CE, T2W and T2-FLAIR sequences to predict response to EGFR-TKI treatment. Similarly, the RS-BM-COM were established based on BM. Finally, the VPE was incorporated into the RS-BTI-COM as a clinical model, resulting in a new model that integrates both radiomics features and VPE, referred to as RS-BTIplusVPE.

### 2.6 Statistical analysis

**FIGURE 2:**
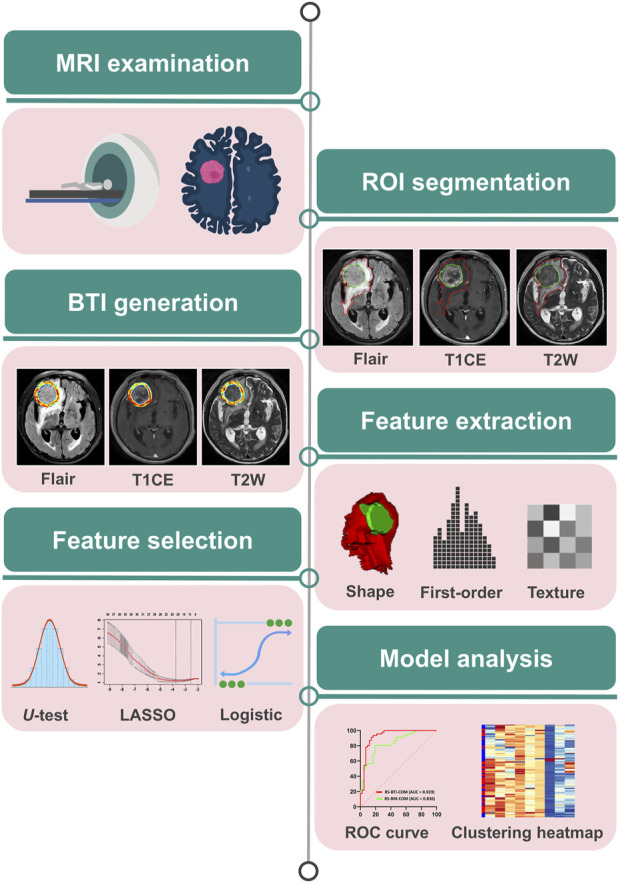
Overview of radiomics analysis workflow.

Clinical factors between responders and non-responders were statistically analyzed using SPSS (version 27.0) and R language (version 4.2.2). The Mann-Whitney U test and chi-square test were used for continuous and categorical variables, respectively. A p-value of less than 0.05 was considered statistically significant. Receiver Operating Characteristic (ROC) curves and Area Under Curve (AUC) were used to assess the ability of features to predict response to EGFR-TKIs. Delong’s test (DeLong et al., 1988) was used to compare differences in AUC values of the models. Figure 2 shows the study design. This study was approved by the ethics committee on 23 August 2022.

## 3 Results

### 3.1 Patient characteristics

Table 1 listed the demographic and clinical characteristics of all patients with BM from NSCLC in both centers. There was no statistical significance in age, gender, smoking status and performance status (PS) of the patients. While, for predicting response to EGFR-TKI, a significant difference (*p* < 0.05) in VPE was found between the responder and non-responder groups across all three cohorts, which suggests that VPE may be correlated with patients’ responses to EGFR-TKI treatment (Fan et al., 2023b).

**TABLE 1:** Clinical characteristics of the study population for predicting response to EGFR-TKI.

Characteristic	Training cohort (n = 88)			Internal validation cohort (n = 45)			External validation cohort (n = 52)		
	Responder (n = 46)	Non-responder (n = 42)	*p*	Responder (n = 23)	Non-responder (n = 22)	*p*	Responder (n = 26)	Non-responder (n = 26)	*p*
Age (Mean ± SD)	61.04 ± 9.47	59.64 ± 9.17	0.464	63.65 ± 11.13	61.68 ± 11.2	0.296	60.31 ± 10.03	57.2 ± 8.67	0.551
Gender			0.608			0.493			0.163
Male	20 (43.5%)	16 (38.1%)		9 (39.1%)	11 (50.0%)		12 (53.8%)	17 (65.4%)	
Female	26 (56.5%)	26 (61.9%)		14 (60.9%)	11 (50.0%)		14 (46.2%)	9 (34.6%)	
Smoking status			0.311			0.848			0.999
Yes	13 (28.3%)	8 (19.0%)		9 (39.1%)	8 (36.4%)		10 (38.5%)	10 (38.5%)	
No	33 (71.7%)	34 (81.0%)		14 (60.9%)	14 (63.6%)		16 (61.5%)	16 (61.5%)	
PS Score			0.635			0.038*			0.271
<2	33 (71.7%)	32 (76.2%)		15 (65.2%)	20 (90.9%)		23 (88.5%)	20 (76.9%)	
≥2	13 (28.3%)	10 (23.8%)		8 (34.8%)	2 (9.1%)		3 (11.5%)	6 (23.1%)	
VPE	11.3 ± 6.12	16.38 ± 6.91	0.001*	10.54 ± 7.08	14.79 ± 7.79	0.046*	14.33 ± 8.53	19.23 ± 7.08	0.005*

SD, standard deviation; PS, performance status; VPE, volume of peritumoral edema; *, *p* < 0.05.

### 3.2 Prediction performance of the multi-sequence radiomics signatures

A total of nine radiomics features were selected from the BTI region as the most predictive features with LASSO logistic regression. Supplementary Material 2 shows the process of LASSO-based selection of the features (Supplementary Figure S2). From T1CE, T2W and T2-FLAIR MRI, there were four, one and four features were identified as the most predictive features and used to establish the combined radiomics models (RS-BTI-COM). Formulas of the developed radiomics models were listed in Supplementary Material 1.

**FIGURE 3:**
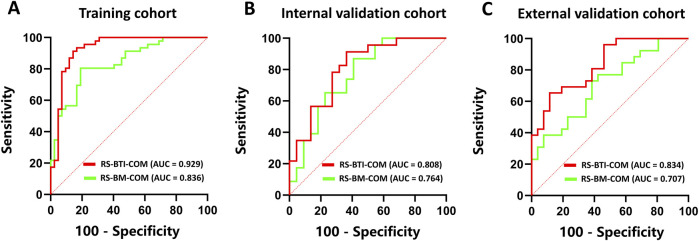
ROC curves of RS-BTI-COM and RS-BM-COM illustrating the performance of radiomics models for predicting response to EGFR-TKI in the training **(A)**, internal validation **(B)**, and external validation **(C)** cohorts.

Table 2 compares predictive performance of the constructed RSs based on the BTI and BM region. The multi-sequence fused RS-BTI-COM achieves higher AUC, SEN, SPE and ACC compared with RS-BM-COM. A possible explanation is that the invasion of metastatic brain tumor cells involves interactions with the brain microenvironment, including changes in astrocytes and vascular structures. The invasive process may lead to dynamic alterations of the BTI, which could be associated with the response to EGFR-TKI (Lorger and Felding-Habermann, 2009). Figure 3 depicted ROC curves of the established RS-BTI-COM and RS-BM-COM.

**TABLE 2:** Performance of the developed models based on BM and BTI for determining response to EGFR-TKI.

RSs	Training cohort				*p*	Internal validation cohort				*p*	External validation cohort				*p*
	AUC (95%CI)	SEN	SPE	ACC		AUC (95% CI)	SEN	SPE	ACC		AUC (95% CI)	SEN	SPE	ACC	
RS-BTI-COM	0.929 (0.870–0.989)	0.913	0.857	0.841		0.808 (0.680–0.936)	0.913	0.636	0.756		0.834 (0.728–0.941)	0.885	0.654	0.673	
RS-BM-COM	0.836 (0.754–0.919)	0.804	0.810	0.761		0.765 (0.623–0.907)	0.870	0.591	0.711		0.707 (0.566–0.848)	0.769	0.577	0.615	
RS-BTI-COM vs. RS-BM-COM					0.069					0.645					0.144

AUC: area under the receiver operating characteristic curve; CI: confidence interval; SEN: sensitivity; SPE: specificity; ACC: accuracy.

### 3.3 Prediction performance of the combined model

**FIGURE 4:**
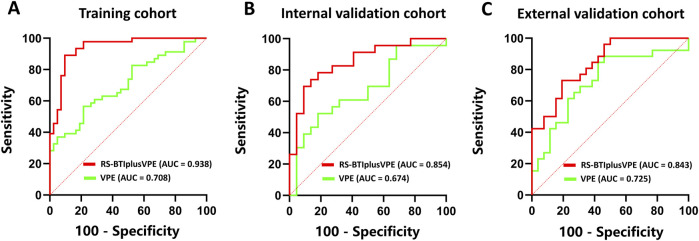
ROC curves of VPE and RS-BTIplusVPE in the training **(A)**, internal validation **(B)** and external validation **(C)** cohorts.

The RS-BTI-COM was then integrated with VPE to establish the RS-BTIplusVPE. Table 3 compared performances of the VPE, RS-BTI-COM and RS-BTIplusVPE. When the VPE was used alone to predict response to EGFR-TKI, the AUCs yielded were ranged from 0.674 to 0.725 in primary and external cohorts. The RS-BTIplusVPE exhibited the highest AUCs (0.843–0.938), SPE (0.808–0.905) and ACC (0.712–0.875). Delong’s test indicates a significant difference (p < 0.05) between VPE and RS-BTIplusVPE in both the training and internal validation cohorts. These results demonstrated that VPE may serve as a clinical indicator, providing complementary information for BTI-based radiomic models. Figure 4 depicted ROC curves of the VPE and RS-BTIplusVPE.

**TABLE 3:** Performance of VPE, RS-BTI-COM and combined models for determining the response to EGFR-TKI.

RSs	Training cohort				*p*	Internal validation cohort				*p*	External validation cohort				*p*
	AUC (95%CI)	SEN	SPE	ACC		AUC (95% CI)	SEN	SPE	ACC		AUC (95% CI)	SEN	SPE	ACC	
RS-BTI-COM	0.929 (0.870–0.989)	0.913	0.857	0.841		0.808 (0.680–0.936)	0.913	0.636	0.756		0.834 (0.728–0.941)	0.885	0.654	0.673	
VPE	0.708 (0.601–0.816)	0.565	0.786	0.636		0.674 (0.513–0.835)	0.522	0.818	0.644		0.725 (0.582–0.868)	0.885	0.538	0.635	
RS-BTIplusVPE	0.938 (0.888–0.989)	0.891	0.905	0.875		0.854 (0.742–0.966)	0.696	0.909	0.778		0.843 (0.740–0.946)	0.731	0.808	0.712	
RS-BTI-COM vs RS-BTIplusVPE					0.526					0.330					0.596
VPE vs RS-BTIplusVPE					0.0001*					0.022*					0.081

AUC: area under the receiver operating characteristic curve; CI: confidence interval; SEN: sensitivity; SPE: specificity; ACC: accuracy.

### 3.4 Radiomics features analysis

A total of nine radiomics features were identified as key predictors for determining the response to EGFR-TKI treatment. Among these, eight features were classified under the textural category, reflecting complex patterns within the image data, while only the remaining one features belonged to the first-order category, capturing essential statistical information from the voxel intensity distributions. These selected features play a crucial role in differentiating between responders and non-responders to EGFR-TKI therapy. Detailed performance metrics of the identified features are summarized in Table 4. The detailed meanings of the identified features are provided in Supplementary Material 1. Supplementary Material 2 shows cluster analyses of the selected radiomics features (Supplementary Figure S3).

**TABLE 4:** Performance metrics of the selected features for predicting EGFR-TKI response in NSCLC patients with BM in the training cohort.

Feature	Sequence	Weighting coefficient	Mean ± SD		AUC	*p*
			Responder	Non-responder		
Wavelet-HHH_glrlm_HighGrayLevelRunEmphasis (F1)	T2-FLAIR	−24.698193	−0.23 ± 1.11	0.25 ± 0.81	0.674	0.003*
Wavelet-HHL_glcm_SumEntropy (F2)	T2-FLAIR	44.793039	0.21 ± 0.84	−0.23 ± 1.12	0.624	0.044*
Wavelet-HLH_glcm_ClusterShade (F3)	T2-FLAIR	−5.974253	−0.22 ± 1.17	0.24 ± 0.72	0.641	0.018*
Wavelet-LHH_firstorder_Mean (F4)	T2-FLAIR	1.185793	0.21 ± 0.95	−0.23 ± 1.01	0.641	0.019*
lbp-3D-k_glszm_GrayLevelNonUniformityNormalized (F5)	T1CE	7.918925	0.23 ± 0.97	−0.25 ± 0.99	0.649	0.015*
Wavelet-HHH_firstorder_Uniformity (F6)	T1CE	1,679.037251	0.23 ± 0.86	−0.25 ± 1.09	0.643	0.015*
Wavelet-HHH_glcm_SumAverage (F7)	T1CE	−15.838085	−0.21 ± 0.12	0.23 ± 1.42	0.631	0.027*
Wavelet-HLH_glcm_MCC (F8)	T1CE	−11.903183	−0.22 ± 0.74	0.24 ± 1.19	0.636	0.027*
Log-sigma-5-0-mm-3D_gldm_SmallDependenceEmphasis (F9)	T2W	165.294229	0.26 ± 1.12	−0.28 ± 0.77	0.644	0.015*

SD, standard deviation; *, *p* < 0.05.

## 4 Discussion

NSCLC is the most common subtype of lung cancer, therefore, early assessment of the response to targeted therapy can significantly benefit the personalized treatment of NSCLC patients (Ai et al., 2018). Most previous studies have focused on predicting the efficacy of EGFR-TKI therapy only based on the primary lung tumor (Wang et al., 2019; Zhao et al., 2017; Chen et al., 2022; Xu et al., 2019). Fan et al. (Fan et al., 2022; Fan et al., 2023b; Fan et al., 2023a) have revealed that important information for predicting the response to EGFR-TKI therapy also exists within BM. However, these studies have primarily concentrated on T1-weighted and T2W MRI sequences. The potential of T2-FLAIR in reflecting the microenvironment and medical mechanisms of brain metastases remains unexplored. In this study, the RS-BTIplusVPE model was developed and validated for the early prediction of the response to EGFR-TKI therapy in NSCLC patients with BM, which integrating BTI radiomics features from T1CE, T2W and T2-FLAIR MRI sequences and VPE. The RS-BTIplusVPE model outperformed BTI-based model, demonstrating that VPE is significantly associated with the response to EGFR-TKI therapy (*p* < 0.05). Additionally,our research confirmed that the BTI region exhibits greater heterogeneity than whole BM area, containing more information that plays a positive role in predicting EGFR-TKI treatment response. Furthermore, we found that incorporating the T2-FLAIR MRI sequence enables the extraction of valuable features from the BTI region more effectively.

BTI is a biologically active zone where tumor cells invade and spread into adjacent brain tissue while interacting with the surrounding brain microenvironment, including immune components (Tabassum et al., 2023). At this interface, tumor cells can also evade immune surveillance by expressing immunosuppressive molecules, such as PD-L1, thereby promoting tumor growth and dissemination (Xiao et al., 2021b). Studies have shown (Li et al., 2023) that the activity of immune cells (e.g., macrophages and T cells) at the tumor margins within the BTI microenvironment can directly impact the patient’s therapeutic prognosis. This interaction between the BTI microenvironment and the host immune system may provide valuable insights into tumor behavior under EGFR-TKI treatment, suggesting that the BTI region holds more predictive value than features derived solely from the tumor mass of BM. Additionally, peritumoral edema is another common feature in the BTI, often associated with pro-inflammatory and angiogenic factors (e.g., VEGF) released by tumor cells, which compromise vascular integrity and lead to fluid leakage into the brain parenchyma (Chen et al., 2024). MRI-based studies (Joo et al., 2021) have indicated that radiomic features of the BTI, such as tumor margin blurriness, are closely related to tumor invasiveness, providing critical information for assessing tumor progression and predicting response to targeted therapies. Our findings also suggest that the BTI in BMs likely contains important information relevant to therapeutic response, further supporting the potential of BTI-based radiomics in improving clinical decision-making for targeted treatments. Further research is needed to deepen our understanding of the biological mechanisms underlying these findings.

The incorporation of the T2-FLAIR sequence in brain MRI is crucial for extracting high-value features from the BTI region. T2-FLAIR is particularly effective at suppressing cerebrospinal fluid signals, thereby enhancing the visibility of peritumoral edema and other abnormal brain tissues (Zhang et al., 2021). Given that peritumoral edema is a common feature in the BTI region, T2-FLAIR allows for the extraction of valuable radiomic features that are difficult to capture using other sequences like T1 or T2-weighted images. Studies have shown that combining T2-FLAIR with radiomic analysis significantly improves the accuracy of predicting tumor invasiveness and treatment response, especially in gliomas and BMs (Dvořák, 2015; Rajkumar and Kavitha, 2011). The enhanced contrast between abnormal and normal brain tissues provided by T2-FLAIR facilitates more precise segmentation and feature extraction in the BTI, which is critical for evaluating the biological behavior of the tumor (Xiao et al., 2021a). Moreover, T2-FLAIR-based analysis of the BTI has been associated with more accurate predictions of therapeutic outcomes. By capturing subtle changes at the tumor-brain interface, T2-FLAIR-derived features offer deeper insights into tumor invasiveness and progression, supporting the development of more personalized treatment strategies (Aboian et al., 2022).

In feature analysis of this study, we found that eight texture features and only one first-order extracted from the BTI region showed significant differences between patients with and without a response to EGFR-TKI therapy (*p* < 0.05). According to the texture feature analysis, we found that four texture features extracted from the gray-level co-occurrence matrix (GLCM) in the BTI region showed significant differences between patients with and without a response to EGFR-TKI therapy (*p* < 0.05). These GLCM-based features reflect the disorder or randomness of intensity values, which are typically associated with varying cellular densities or the presence of different tissue types (e.g., tumor infiltration) (Qin et al., 2017). This finding suggests that the heterogeneity within the BTI region is closely related to the efficacy of targeted therapy observed in this study. Moreover, the only first-order feature extracted from the T2-FLAIR sequence within the BTI region, which reflects the average intensity, effectively reveals the pattern of edema spread and its predictive value for response to EGFR-TKI (Liu et al., 2021). This study also found a correlation between the value of VPE and treatment response (*p* < 0.05) across all three cohorts, suggesting that features from this region could serve as potential predictive markers of therapeutic efficacy.

We assessed the value of VPE in predicting the response to EGFR-TKI therapy in NSCLC patients with brain metastases. While VPE has been recognized as an important imaging marker in differentiating between primary brain tumors and BMs (Baris et al., 2016), its role in predicting therapeutic outcomes remains uncertain. Our analysis showed that although VPE contributed to model performance, its predictive ability was relatively limited compared to other radiomic features (such as those derived from the BTI region). VPE reflects the extent of vasogenic edema surrounding the tumor, which is associated with factors like tumor-induced disruption of the blood-brain barrier and local inflammation (Esquenazi et al., 2017). However, it does not fully capture the biological complexity related to EGFR-TKI response. This suggests that while VPE is a useful imaging marker for assessing tumor burden and edema, its contribution to predicting EGFR-TKI response is limited, and further research is needed to explore its underlying biological mechanisms.

This study has several limitations. First, the retrospective nature of the study and the limited sample size necessitate further validation of the model using prospective data. Second, although the BTI region were derived from MR images, pathological confirmation was not performed due to the study’s retrospective design. Third, while BTI region segmentation was automated, tumor and peritumoral edema segmentation was done manually, introducing potential subjective bias. Fourth, future work will focus on exploring the correlations between MRI-based morphological variations and histological microstructure to enhance the intratumoral partitioning algorithm. Fifth, although this study suggests a potential correlation between the 4 mm BTI and the efficacy of EGFR-TKI treatment, it lacks the determination of the optimal expansion distance for generating the BTI ROI. Lastly, future studies should further investigate the relationship between tumor heterogeneity in brain tissue and EGFR-TKI treatment response, aiming to explore the underlying biological mechanisms and enhance the interpretability of radiomics models.

## 5 Conclusion

In conclusion, our study developed a validated non-invasive model (RS-BTIplusVPE) by integrating multi-sequence radiomic model and VPE, which showed improved prediction of EGFR-TKI response in NSCLC patients with brain metastases compared to tumor-focused models. Our findings were validated with clinically obtained data from two centers, which may indicate good potential of our model for assisting in clinical decision-making.

## Data Availability

The data analyzed in this study is subject to the following licenses/restrictions: The datasets generated and/or analysed during the current study are not publicly available due to restrictions but are available from the corresponding authors on reasonable request. Requests to access these datasets should be directed to WJ, xiaoya83921@163.com.
