# Supplementary material for: FLAIR-based radiomics signature from brain-tumor interface for early prediction of response to EGFR-TKI therapy in NSCLC patients with brain metastasis
Source: Front Cell Dev Biol. 2025 May 14;13:1525989. doi: 10.3389/fcell.2025.1525989 (PMC12116672; doi:10.3389/fcell.2025.1525989)
Supplement: Supplementary file 1 [file DataSheet2.docx]

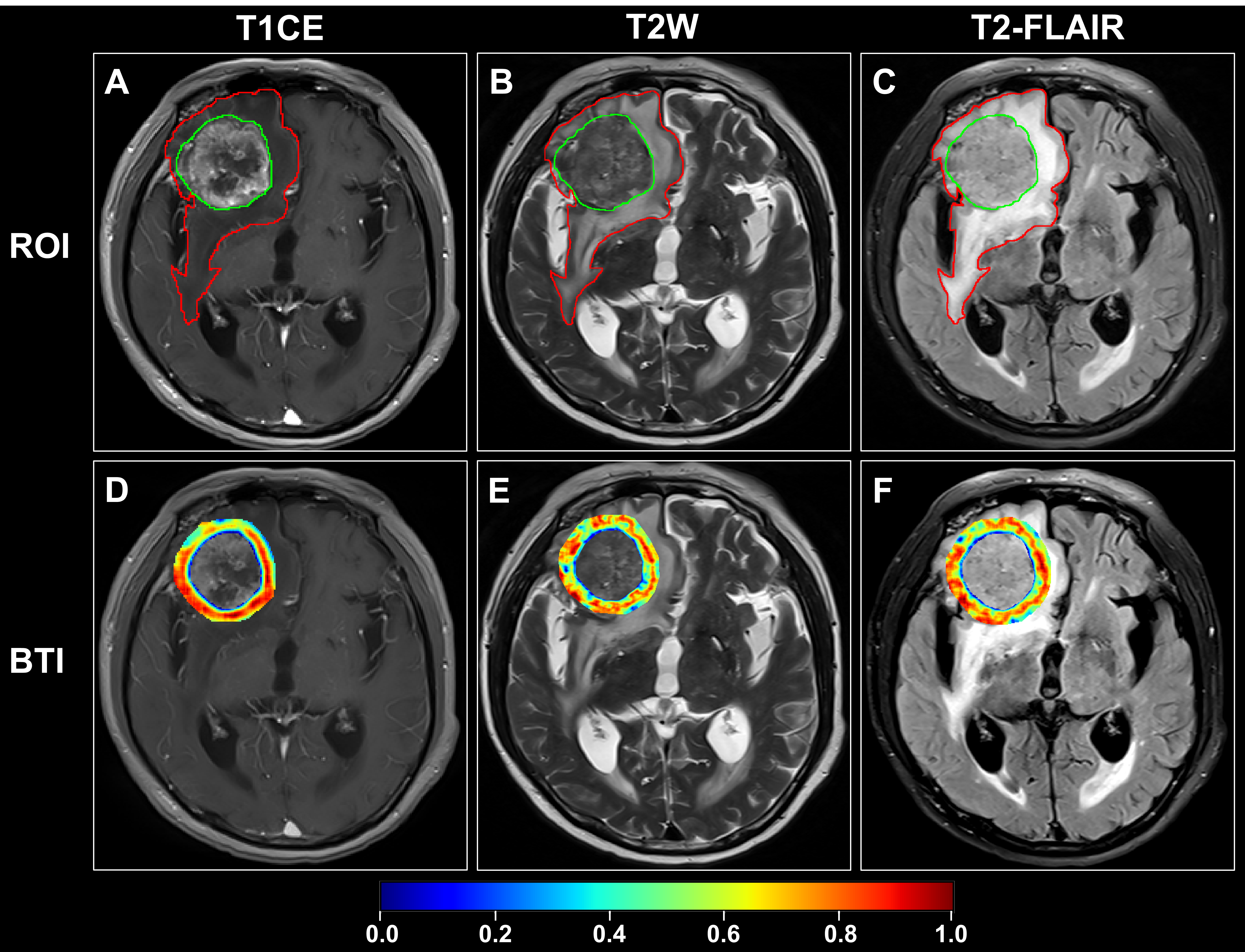


**Figure S1.** Representative Brain MR Images. A 74-year-old male patient who underwent T1CE (A, D), T2W (B, E), and T2-FLAIR (C, F) scans. The top row (A, B, C) displays the segmented ROIs highlighted in red for PEA and green for BM. The bottom row (D, E, F) illustrates the entropy map of BTI, where blue represents lower entropy and red represents higher entropy.


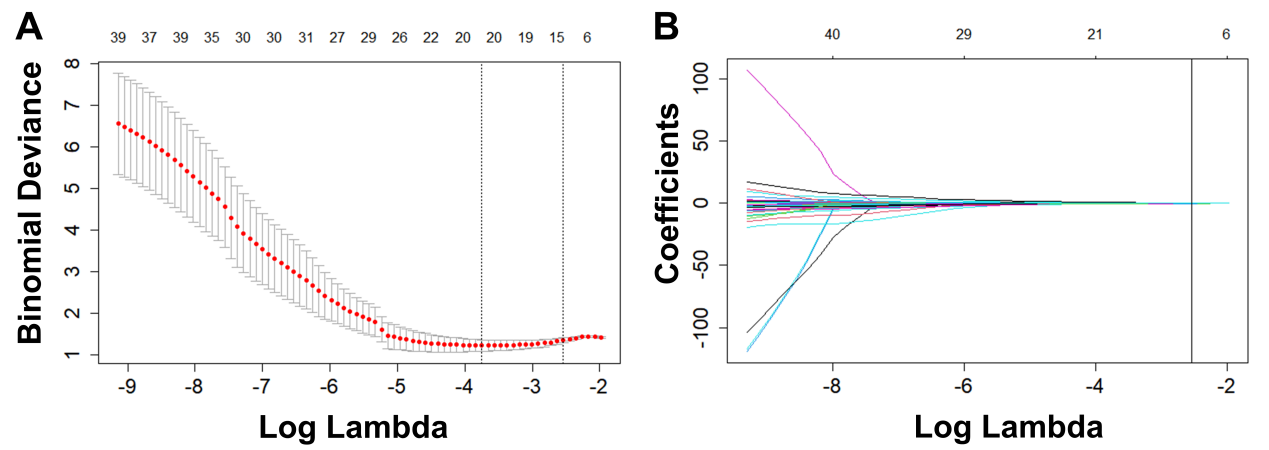


**Figure S2.** Radiomics feature selection using the LASSO binary logistic regression model. (A) Tuning parameter (λ) selection using 10-fold cross-validation, with binomial deviance plotted against log(λ). (B) Coefficient profiles of radiomics features.


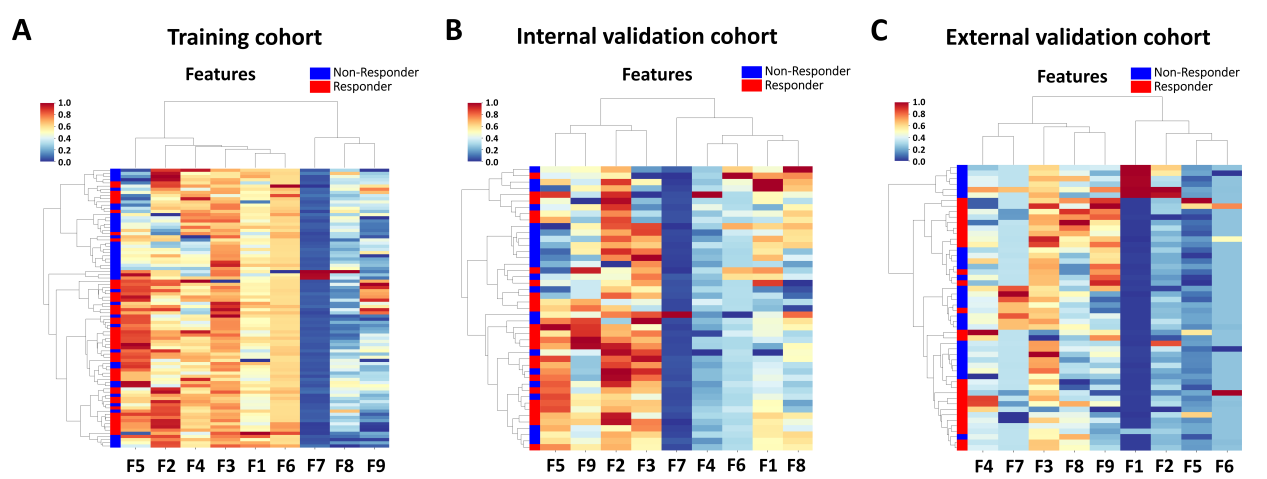


**Figure S3.** Heatmaps showing radiomic feature distributions in patients with BM. Comparison of responder (red) and non-responder (blue) groups in the training (A), internal validation (B) and external validation (C) cohorts. Each heatmap displays a color-coded representation of feature expression levels, ranging from high (red) to low (blue), with hierarchical clustering applied to organize patient and feature groups
