# Supplementary material for: FLAIR-based radiomics signature from brain-tumor interface for early prediction of response to EGFR-TKI therapy in NSCLC patients with brain metastasis
Source: Front Cell Dev Biol. 2025 May 14;13:1525989. doi: 10.3389/fcell.2025.1525989 (PMC12116672; doi:10.3389/fcell.2025.1525989)
Supplement: Supplementary file 2 [file DataSheet1.docx]

**MRI scans in Center 1**

The scanning parameters of contrast-enhanced T1-weighted (T1CE) sequence at 3.0 T MRI were as follows: FOV = 230 × 230 mm, slice thickness = 6.5 mm, Repeat time (TR) = 180 ms, Echo time (TE) = 1.3 ms and matrix size = 256 × 256. The scanning parameters of T2-weighted (T2W) sequence at 3.0 T MRI were as follows: FOV = 230 × 230 mm, slice thickness = 6.5 mm, TR = 2000 ms, TE = 80 ms and matrix size = 256 ×256 mm. The scanning parameters of T2 fluid-attenuated inversion recovery (T2-Flair) sequence were as follows: FOV = 230 × 230 mm, slice thickness = 6.5 mm, TR = 7000 ms, TE = 125 ms and matrix size = 208 × 161 mm.

**MRI scans in Center 2**

The scanning parameters of T1CE sequence at 3.0 T MRI were as follows: FOV = 194 × 230 mm, slice thickness = 5mm, TR = 270 ms; TE = 2.48 ms and matrix size = 320 × 216 mm. The scanning parameters of T2W sequencewere as follows: FOV = 194 × 230 mm, slice thickness = 5 mm, TR = 3630 ms, TE = 87ms and matrix size = 384 × 227 mm. The scanning parameters of T2-Flair sequence at 3.0 T MRI were as follows: FOV = 194 × 230 mm, slice thickness = 5 mm, TR = 7800 ms, TE = 165 ms and matrix size = 384 × 227 mm.

**The formula for calculating the entropy**

After obtaining the ROI of the BTI region, we calculate the entropy value of the BTI region to quantify the entropy of the pixel intensity distribution within the region, reflecting its texture complexity and information content. The following is the formula for calculating the entropy of image pixels:

$$H(X)=-\sum_{i=1}^{n} p(x_{i})log(p(x_{i}))$$

**ICC Calculation Methods**

ICC (1, 1): This was used to evaluate the consistency of measurements from multiple scans of the same subject at different time points (test-retest consistency), representing the stability of radiomic features when a single physician delineates the region of interest (ROI) multiple times (intra-ICC).

ICC (3, 1): This was used to evaluate the consistency of radiomic features between different observers (inter-observer consistency), reflecting the agreement of different physicians when delineating the same ROI (inter-ICC).

**Detailed meanings of the identified features**

(1) wavelet-HHH_glrlm_HighGrayLevelRunEmphasis (F1): A feature from the Gray-Level Run Length Matrix (GLRLM) that measures the emphasis on high gray-level runs in the image. This indicates how much the image contains long, continuous runs of high gray levels, which can be associated with tumor heterogeneity.

(2) wavelet-HHL_glcm_SumEntropy (F2): A feature from the Gray-Level Co-occurrence Matrix (GLCM) that quantifies the entropy, or randomness, of the image's gray-level distribution. This can reflect the degree of texture complexity in the tumor.

(3) wavelet-HHL_glcm_ClusterShade (F3): A feature from GLCM that assesses the local linear dependence of gray levels in the image. It provides information about the spatial arrangement of the pixels and how they contribute to the overall image structure.

(4) wavelet-LHH_firstorder_Mean (F4): A first-order statistical feature that measures the mean intensity value of the image. This is a fundamental feature to assess the overall intensity distribution of the tumor.

(5) lbp-3D-k_glszm_GrayLevelNonUniformityNormalized (F5): A feature from the Gray-Level Size Zone Matrix (GLSZM) that quantifies the non-uniformity of the gray levels in the image. It captures how unevenly the intensity is distributed across the tumor and surrounding regions.

(6) wavelet-HHH_firstorder_Uniformity (F6): A first-order statistical feature that measures the uniformity or smoothness of the image's intensity distribution. This feature can help assess the homogeneity of the tumor and surrounding tissue.

(7) wavelet-HHL_glcm_SumAverage (F7): A feature from GLCM that measures the average of all possible gray-level pair combinations. It quantifies the general gray-level interaction in the image.

(8) wavelet-HLH_glcm_MCC (F8): A feature from GLCM that quantifies the maximum correlation coefficient. It captures the level of correlation between adjacent pixels and can help assess tumor texture.

(9) log-sigma-5-0-mm-3D_gldm_SmallDependenceEmphasis (F9): A feature from the Gray-Level Dependence Matrix (GLDM) that focuses on small dependence emphasis in the image. This feature highlights areas of the image where pixel values are highly dependent on their neighbors, which is indicative of fine-grained texture features.
